# Serum Albumin and Post-Stroke Outcomes: Analysis of UK Regional Registry Data, Systematic Review, and Meta-Analysis

**DOI:** 10.3390/nu16101486

**Published:** 2024-05-14

**Authors:** Rosa J. Thuemmler, Tiberiu A. Pana, Ben Carter, Ribeya Mahmood, Joao H. Bettencourt-Silva, Anthony K. Metcalf, Mamas A. Mamas, John F. Potter, Phyo K. Myint

**Affiliations:** 1 Institute of Applied Health Sciences, School of Medicine, Medical Sciences and Nutrition, University of Aberdeen, Aberdeen AB24 3FX, UK; r.mahmood.20@abdn.ac.uk (R.M.); phyo.myint@abdn.ac.uk (P.K.M.); 2 Aberdeen Cardiovascular and Diabetes Centre, Institute of Medical Sciences, University of Aberdeen, Aberdeen AB24 3FX, UK; tiberiu.pana@abdn.ac.uk; 3 Department of Biostatistics and Health Informatics, Institute of Psychiatry, Psychology and Neuroscience, King’s College London, London WC2R 2LS, UK; ben.carter@kcl.ac.uk; 4 Norwich Medical School, University of East Anglia, Norwich NR4 7TJ, UK; joao.bettencourt@nnuh.nhs.uk (J.H.B.-S.); john.potter@uea.ac.uk (J.F.P.); 5 Stroke Research Group, Norfolk and Norwich University Hospital, Norwich NR4 7UY, UK; kneale.metcalf@nnuh.nhs.uk; 6 Keele Cardiovascular Research Group, Centre for Prognosis Research, Institute for Primary Care and Health Sciences, Keele University, Stoke-on-Trent ST5 5BG, UK; mamasmamas1@yahoo.co.uk

**Keywords:** ischaemic stroke, albumin, long-term mortality, in-hospital outcomes, nutritional

## Abstract

Hypoalbuminemia associates with poor acute ischemic stroke (AIS) outcomes. We hypothesised a non-linear relationship and aimed to systematically assess this association using prospective stroke data from the Norfolk and Norwich Stroke and TIA Register. Consecutive AIS patients aged ≥40 years admitted December 2003–December 2016 were included. Outcomes: In-hospital mortality, poor discharge, functional outcome (modified Rankin score 3–6), prolonged length of stay (PLoS) > 4 days, and long-term mortality. Restricted cubic spline regressions investigated the albumin–outcome relationship. We updated a systematic review (PubMed, Scopus, and Embase databases, January 2020–June 2023) and undertook a meta-analysis. A total of 9979 patients were included; mean age (standard deviation) = 78.3 (11.2) years; mean serum albumin 36.69 g/L (5.38). Compared to the cohort median, albumin < 37 g/L associated with up to two-fold higher long-term mortality (HR_max_; 95% CI = 2.01; 1.61–2.49) and in-hospital mortality (RR_max_; 95% CI = 1.48; 1.21–1.80). Albumin > 44 g/L associated with up to 12% higher long-term mortality (HR_max_1.12; 1.06–1.19). Nine studies met our inclusion criteria totalling 23,597 patients. Low albumin associated with increased risk of long-term mortality (two studies; relative risk 1.57 (95% CI 1.11–2.22; *I*^2^ = 81.28)), as did low-normal albumin (RR 1.10 (95% CI 1.01–1.20; *I*^2^ = 0.00)). Strong evidence indicates increased long-term mortality in AIS patients with low or low-normal albumin on admission.

## 1. Introduction

Serum albumin has been shown to play a neuroprotective role in acute stroke in pre-clinical studies [1]. It is the primary modulator of oncotic pressure, opposing the effects of hydrostatic blood pressure and maintaining intravascular volume, has potent antioxidant and anti-inflammatory properties, and inhibits platelet aggregation. These factors may therefore contribute to limiting cerebral ischaemia and oedema at the time of cerebral infarction, resulting in improved outcomes [1,2]. Albumin is also an important marker of nutritional status and inflammatory burden, both mediators of adverse stroke outcomes [3].

Serum albumin levels have been shown to be a predictor of stroke outcomes. A recent national prospective study from China including >13,000 patients concluded that low albumin levels were related to poor functional outcome and higher mortality after acute ischemic stroke (AIS) and transient ischemic attack (TIA) [4]. Other small-scale observational studies exist but utilize a small participant sample size, lack appropriate confounding, and model the relationship as a linear dose–response relationship [5,6,7,8,9]. Whilst studies have looked at albumins’ short- and medium-term outcomes, albumins’ association with long-term outcomes remains unknown. Additionally, further large scales studies are required to confirm findings suggestive of a non-linear relationship between albumin and post-stroke outcomes.

Against this background, we aimed to conduct primary analyses to determine the association of albumin levels with stroke functional outcomes and mortality including a large prospective cohort of unselected stroke patients with long-term follow-up. Furthermore, we also aimed to quantify current evidence by conducting a focused systematic review and meta-analysis of albumin levels and clinical stroke outcomes.

## 2. Materials and Methods

### 2.1. Primary Data Analysis

#### Study Design and Data Source

This study was a prospective cohort study and adheres to the observational cohort guidelines (STROBE) [10]. This study used data from the Norfolk and Norwich Stroke and TIA Register (NNSTR); a prospectively maintained stroke register in the United Kingdom that records all stroke admissions to the Norfolk and Norwich University Hospital. The hospital served a catchment area of approximately 1,016,000 in 2018 [11]. The Newcastle and Tyneside National Health Service and Research Ethics Committee provided ethical approval for this database (17/NE/0277), allowing anonymized research studies without individual patients’ consent. This study was conducted in accordance with the principles of the declaration of Helsinki (1964) and later amendments. Full data collection methods have been previously published in detail [12]. Patients were included if they were >40 years old, were admitted with a diagnosis of acute ischaemic stroke (AIS), and had complete follow-up data between their inclusion in this study and the end of the follow-up period in December 2016. Median (IQR) follow-up was 5.5 (3.02–9.20) years.

### 2.2. Data Extraction and Exposures

Baseline patient demographics were extracted from the database, along with relevant covariates and comorbidities. Data extracted were as follows: age, sex, pre-stroke modified Rankin scale (mRS), Oxford Community Stroke Project classification (OCSP), prescription at admission and discharge (anticoagulants and antiplatelets), and stroke-associated pneumonia during admission (SAP), defined as any pneumonia within 7 days of admission [13]. All pre-existing comorbidities recorded in the database were extracted using International Classification of Disease-tenth edition (ICD-10) codes (Supplementary Table S1). Admission levels of albumin, white blood cells (WBC), and C-reactive protein (CRP) blood tests were extracted as the first-ever recorded measurement in the linked registry records after the index AIS admission. The primary exposure of interest were albumin levels at the time of admission (g/L).

### 2.3. Outcomes

All-cause mortality following admission was the primary outcome, and the following secondary outcomes were included: functional outcome and prolonged length of stay (PLoS) which was defined as a length of stay in hospital for >4 days. Functional outcome was assessed at discharge and recorded using the modified Rankin score(mRS). Poor functional outcome was defined as mRS of 3–6 (disability/death).

### 2.4. Missing Data and Exclusions

Missing data were handled in a predefined manner. Variables with ≥5% missing data (albumin, NIHSS, mRS on admission and at discharge, OCSP classification, CRP, and WBC) were imputed. Amongst the 10,636 admissions, patients were excluded if they had missing follow-up (*n* = 79), missing date of discharge (*n* = 22), if their first albumin measurement occurred after discharge (*n* = 371), or if they died on the day of admission (*n* = 33). Table S2a–g in the Supplementary Materials, summarises key variables with missing data after applying the selection criteria: mRS before incident AIS (5.35%), albumin (3.88%), OCSP classification (9.76%), NIHSS (89.59%), CRP (16.85%), and WBC (2.06%). Missing data analysis revealed that patients with missing data on these variables were less likely to have pre-comorbidities, received less in-hospital medication and had significantly longer in-hospital stays. Data missingness was therefore likely dependant on observed but not unobserved data and therefore deemed likely missing-at-random [14]. A multiple imputation by chained equation (MICE) algorithm with 20 iterations was employed to impute missing NIHSS, albumin, OCSP, CRP, and WBC values using an ordinal logistic regression with the predictors outlined in Table S3. A separate MICE algorithm was employed for variables utilised for the long-term-mortality analyses, replicating the methodology above but also including the Nelson–Aalen estimator as a predictor. Due to the high degree of missingness of the NIHSS variable, sensitivity analyses not including NIHSS adjustment were undertaken.

### 2.5. Statistical Analyses

Statistical analyses were performed using Stata v14.1. Patient characteristics were compared between albumin quartile groups. Categorical variables were compared using Pearson’s Chi-square test, normally distributed continuous variables using analysis of variance (ANOVA), and non-normally distributed continuous variables using the Kruskal–Wallis test. Significance was set at *p* < 0.05.

### 2.6. Clinical Outcomes

For the in-hospital outcomes (mortality, functional outcome, and prolonged length of stay), we employed Poisson regression models with a robust variance estimator. The use of the robust variance estimator allows the relaxation of the assumption that the outcome follows a Poisson distribution and therefore allows the derivation of risk ratio (RR) with appropriate standard error and an outcome following a binomial distribution [15]. This method was chosen as an alternative to traditional logistic regressions, to yield RR directly comparable to the hazard ratios (HR) from Cox regressions [15]. Cox-regressions were performed to assess long-term mortality post-admission.

Both models were constructed to evaluate the relationship between albumin as a continuous variable and the selected adverse outcomes, adjusted for confounders, including NIHSS. We determined the best-fitting model using Akaike Information Criterion (AIC), considering linear and nonlinear models (restricted cubic splines, RCS) with varying degrees of freedom (*df* = 2 to *df* = 7). RCS were constructed using the Stata command *rcsgen*. Where an RCS model has a lower AIC than the linear model, the likelihood-ratio test was used to confirm that this RCS model provides a better fit for the data than the linear model. Ultimately, albumin was parametrized using RCS with 3 degrees of freedom (2 internal knots) for PLoS and poor functional outcome and with 4 degrees of freedom (3 internal knots) for short- and long-term mortality. The reference point (HR/RR = 1) was chosen as the minimum value for all functions, corresponding to the median albumin value of 37 g/L. We assessed proportional hazards for Cox regression models with Schoenfeld residuals.

Finally, we conducted regressions categorizing albumin into quartiles (20–34 g/L, 35–37 g/L, 38–41 g/L, 42–47 g/L) to facilitate comparisons with previous studies.

### 2.7. Systematic Review and Meta-Analysis

This review followed the Preferred Reporting Items for Systematic Review and Meta-Analyses (PRISMA) statement and was registered with the International Prospective Register of Systematic Reviews (PROSPERO registration number: CRD42023418592). PubMed, Scopus, and Embase were searched between January 2020 and June 2023. Studies published before January 2020 were sourced from a 2021 systematic review [4]. The search strategy was verified by a medical librarian. The detailed search strategy for the respective databases is detailed in Supplementary Methods S1. Articles were eligible if (1) the subjects were patients with AIS, (2) the design was a prospective study, (3) the exposure was serum albumin level (either categorised or continuous), (4) the outcome was poor functional outcome, mRS, in-hospital- or long-term mortality. Two reviewers (RJT and RM) undertook abstract and full-text screening with a third reviewer adjudicated conflict (TAP). Data extraction was also performed in duplicate with a third reviewer adjudicating. Risk of bias was conducted using Robin-E [16]. We pooled the reported associations (adjusted RR if available) using the random-effects model in STATA16. The exposure categories of interest were albumin levels (<35 g/L, 35–39.9 g/L, >45 g/L) in patients with AIS versus the referent “normal” category (40–44.9 g/L). Heterogeneity was assessed with the *I*^2^ statistic (>50% was indicative of substantial heterogeneity). Publication bias was assessed using funnel plots if there were >10 studies present.

## 3. Results

### 3.1. Database Study

**Figure 1:**
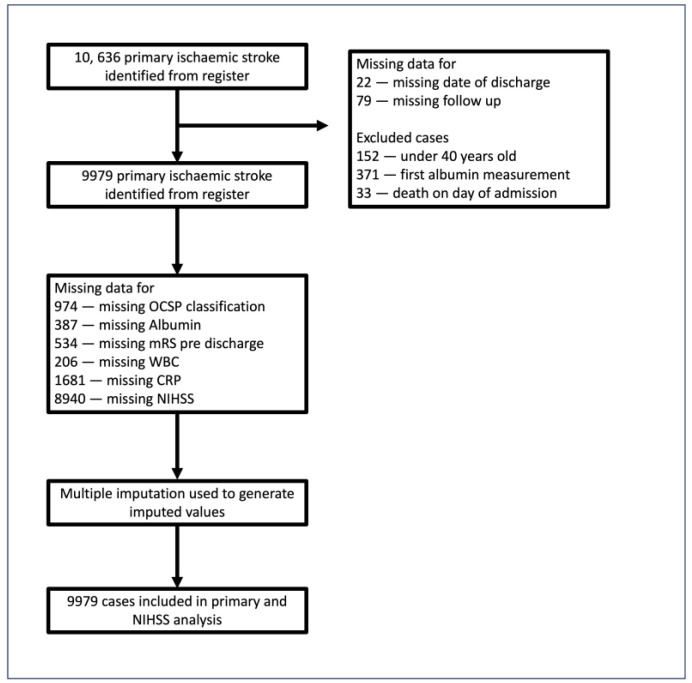
Study population flowchart. Out of 10,636 admissions extracted from the register, the following patients were excluded: those with missing follow-up (*n* = 79) or missing date of discharge (*n* = 22), those for whom the first measurement of albumin recorded in the registry occurred after discharge (*n* = 371), and those who died on the day of admission (*n* = 33).

Of the 10,636 records with primary AIS diagnosis >40 years old, 657 were excluded for reasons outlined in Figure 1. After the imputation of variables with >5% missingness, a total of 9979 cases were used in the final analysis. The sample included in the current study consisted of 9979 patients with acute stroke admitted between December 2003 and December 2016. The mean age (standard deviation) = 78.3 (11.2) years), 57.0% were women, and the mean baseline serum albumin level was 36.69 g/L (5.38), with a median (IQR) of 37 g/L (34–40). Table 1 demonstrates baseline characteristics by admission albumin level, divided into quartiles (<35 g/L, 35–37 g/L, 38–41 g/L, >41 g/L). Overall, 4645 deaths were recorded post-admission, distributed as follows across quartiles: Q1 (1696), Q2 (1035), Q3 (953), and Q4 (961). A Kaplan–Meier curve illustrates this association in Figure S1. Compared with higher serum albumin groups, patients in the lower quartiles were more likely to be older, female, suffer from diabetes and chronic heart disease at baseline, be less independent before onset, and suffer a more severe stroke. Patients with lower albumin levels were significantly more likely to suffer from a total anterior circulation stroke and have significantly higher CRP levels and WBC.

### 3.2. Clinical Outcomes

#### Albumin as a Continuous Variable

**Figure 2:**
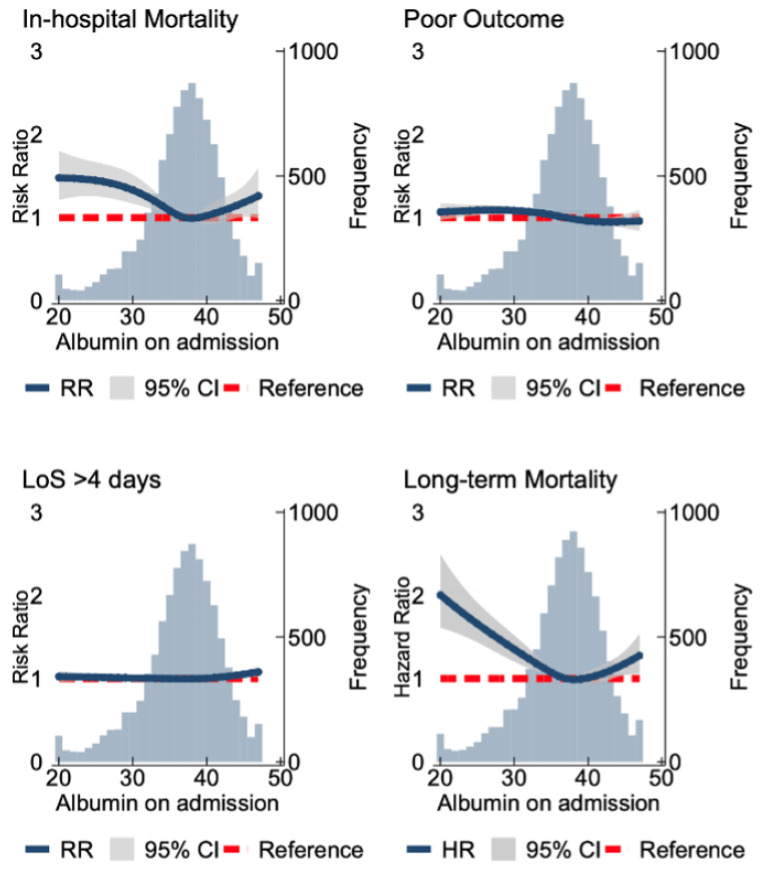
Spline models assessing the association between the serum albumin and clinical outcomes. The association with serum albumin levels and in-hospital mortality, poor functional outcomes (modified Rankin Scale (mRS) score of 3–6), and increased length of stay (>4 days). The association of serum albumin and long-term mortality at 5.5 years follow-up is shown. The blue line indicates the RR/HR, and the grey area indicates the confidence interval. The red line demonstrates the null effect line. The long-term mortality models were fitted by a Cox regression with adjustments made for age, sex, comorbidities (chronic pulmonary disease, atrial fibrillation, cerebrovascular disease, congestive heart disease, heart failure, renal disease, chronic obstructive pulmonary disease, dementia, diabetes, hypertension, hyperlipidemia, liver disease, cancer, peptic ulcer disease, peripheral disease and connective tissue disease, pneumonia (aspiration and non-aspiration)), anti-platelet and anti-coagulation medication on discharge/admission, National Institute of Health Stroke Scale, Oxford Community Stroke Project classification, pre-stroke mRS score, serum white blood count, and C-reactive protein.

Figure 2 details the relationship of albumin and clinical outcomes as a continuous variable. Compared to the cohort median, levels < 37 g/L were significantly associated with up to two-fold higher long-term mortality risk (HR_max_2.01 (95% Confidence Interval 1.61–2.49)) and 48% higher in-hospital mortality (RR_max_1.48 (1.21–1.80)). Compared to the cohort median, levels > 44 g/L were associated with up to 12% higher long-term mortality (HR_max_1.12 (1.06–1.19)) but not in-hospital mortality. There were no associations between admission albumin and PLoS or poor functional outcome. Sensitivity analyses without NIHSS adjustment revealed similar results (Figure S2).

### 3.3. Albumin Quartiles

Analyses by albumin quartiles showed similar results and is further outlined in the Supplementary Materials (Figure S3). Sensitivity analysis without NIHSS adjustment did not attenuate the findings (Figure S4).

### 3.4. Systematic Review and Meta-Analysis

#### Search Results

**Figure 3:**
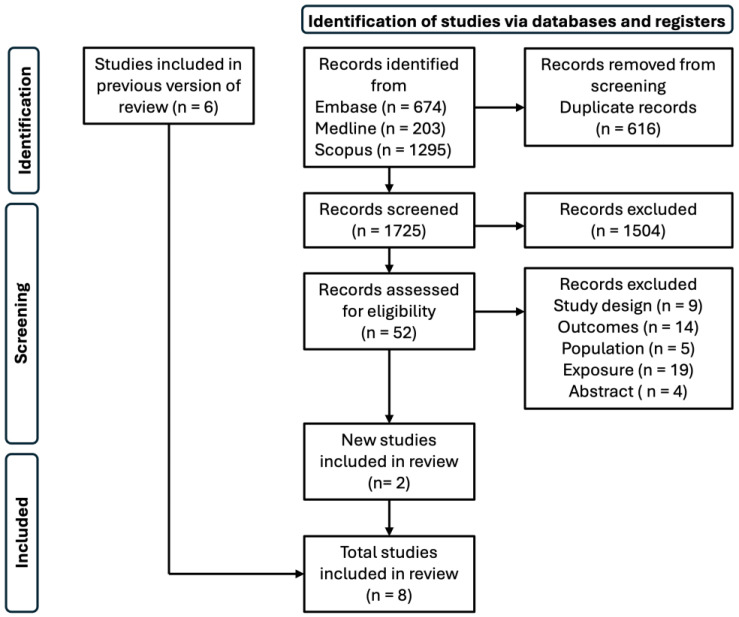
Flow chart of included studies in systematic review.

Figure 3 details the flowchart of the included studies. We identified 2172 studies for inclusion, of which 616 studies were excluded due to duplicity and 1504 were removed due to irrelevancy. After full-text screening of 52 studies, 28 studies were excluded for not conforming to our prespecified criteria: 9 for study design, 14 for outcome, 5 for population, 19 for exposure, and 4 for lacking full text. From our search, two studies were included, including our own [17]. A previous meta-analysis added seven studies: three for functional outcomes [4,6,9] and five for long-term mortality [4,5,7,8,18]. A table outlining all the papers included in the systematic review can be found in the Supplementary Materials (Table S4).

### 3.5. Meta-Analysis

#### Long-Term Mortality

**Figure 4:**
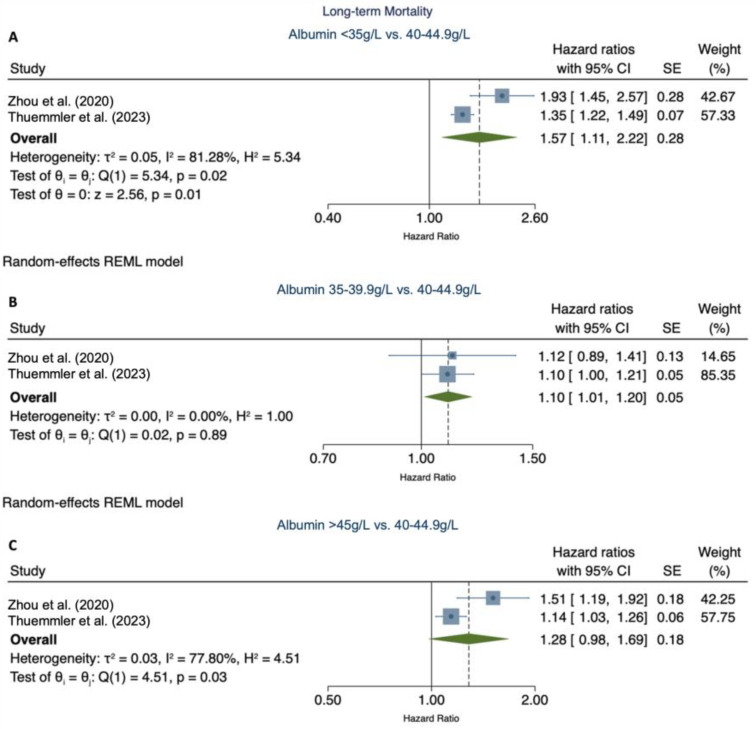
Meta-analysis of the association of categorical serum albumin levels with long-term mortality [4]. (**A**) demonstrates the pooled relative risk of long-term mortality for serum albumin levels < 35 g/L vs. 40–44 g/L. (**B**) demonstrates the pooled relative risk of serum albumin levels 35–39.9 g/L vs. 40–44 g/L. (**C**) demonstrates the relative risk of serum albumin levels > 45 g/L vs. 40–44 g/L. CI, confidence interval; SE, standard error; p, *p*-value.

Two [4] out of six studies [5,7,8,18], including our primary study, were eligible for the meta-analysis. The pooled relative risk of serum albumin levels < 35 g/L and 35–39.9 g/L were associated with increased long-term mortality (pooled RR 1.57 (95% CI 1.11–2.22)) (Figure 4A) and (RR 1.10 (1.01–1.20)) (Figure 4B), respectively. Serum albumin levels >45 g/L were not in association with increased mortality (RR 1.28 (0.98–1.69)) (Figure 4C).

### 3.6. Functional Outcome

Four [4,6,9] out of five studies [4,6,9,17] assessing the risk of poor functional outcomes and serum albumin were eligible for the meta-analysis, including our primary analysis. The pooled relative risk of serum albumin levels < 35 g/L was associated with worse functional outcome (pooled RR 1.49 (1.32–1.67)) (Figure S5A). Serum albumin 35–39.9 g/L also demonstrated significant association, albeit slightly lower (RR 1.12 (1.02–1.24)) (Figure S5B). Serum albumin > 45 g/L showed no association with functional outcome (RR 0.85 (0.69–1.06)). This analysis yielded high evidence of statistical heterogeneity (*I*^2^ = 65.10%). Visual inspection revealed Dziedzic et al. to be an outlier [6]. After excluding this study in a sensitivity analysis, the evidence of heterogeneity decreased to moderate (*I*^2^ = 40.51%). The pooled effect size changed insignificantly (RR 0.93 (0.79–1.09)). Sensitivity analysis was carried out for all three models by excluding our primary analysis, to account for potential heterogeneity arising from varying follow-up times (Figure S6).

Further information on the statistical analysis of the meta-analysis, including details on the exclusion and inclusion of studies can be found in Supplementary Methods S2.

### 3.7. Validity Assessment

**Figure 5:**
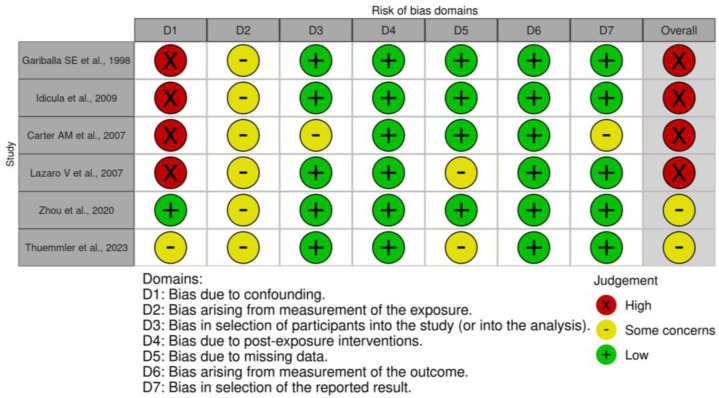
Risk of bias assessment of studies assessing albumin levels and long-term mortality [4,5,7,8,18].

The overall risk of bias in the included studies is summarised in Figure 5 for long-term mortality and Figure S7 for functional outcome. Six studies [5,6,7,9,17] were considered to have high risk of bias, due to confounding. Carter et al. also had high risk of bias due to missing data [18]. All studies denoted some concern of bias related to the fluctuating nature of post-stroke albumin levels and reliance on a single exposure reading. Two studies raised concern of participation bias: Yang et al. [17] only analysed patients who had not received thrombolysis therapy, while Carter et al. [18] recruited white European patients, limiting generalizability. Three studies had some concern of bias due to missing data; our study imputed data, Yang et al. [17] did not outline missing data for the analysis of patients without IVT, and Lazaro et al. did not address missing data in albumin analyses [8]. Three studies [4,6,17] had some concern of bias arising from outcome measurement, due to inadequate information regarding outcome assessors blinding, particularly for functional outcome analysis, as the mRS scale requires judgment. One study has some concern of bias arising from the reported study, this was due to the omission of Q3s albumin effect size on mortality risk. Overall, four [5,7,18] of the six studies [4,5,7,18] in the long-term mortality analysis had a high risk of bias, and two [6,17] of five studies [4,6,9,17] had a high risk of bias in the functional outcome analysis.

## 4. Discussion

In this large-scale hospital-based cohort study, we demonstrated significantly worse long-term and in-hospital outcomes of AIS patients with hypoalbuminemia, including low-normal albumin levels, on admission. Patients with albumin < 37 g/L, had up to 48% increased risk of mortality in-hospital and up to two-fold risk of long-term mortality. Our study confirms the hypothesis of a non-linear relationship between albumin and clinical outcomes. Furthermore, our systematic review and meta-analysis, provides the most comprehensive analysis to date and is the first to report pooled effect sizes of albumin per quartile group, providing a nuanced understanding of its association with post-stroke outcomes.

The relationship of low albumin and worse outcomes was consistent across all the included studies [4,5,6,7,8,9,18]. Whilst this association is often confounded by albumin as an acute-phase reactant, our study demonstrates a short- and long-term association of serum albumin with mortality, independent of inflammatory markers [19]. Another important contributing factor to worse outcomes in patients with hypoalbuminemia is malnutrition [3,20]. Zhou et al., however, adjusted for BMI, a criterion of the diagnosis, and did not find any attenuation of effect after adjustment [4]. This contributes to the evidence of hypoalbuminemia as an independent prognostic indicator of worse AIS outcomes.

Albumin has multifaceted intravascular effects by which it confers neuroprotection [21,22,23,24]. Importantly, it maintains the oncotic pressure of blood and influences the physiological function of the circulatory system [22]. Additionally, it transports fatty acids (FFAs) and replenishes FFAs lost from cellular membranes. Albumin also plays an important role in sustaining neuronal metabolism, under pathological conditions, by increasing the export of pyruvate to neurons [23] and has potent antioxidant properties, through its thiol groups [21]. Albumin impacts the bioavailability of prostacyclin (PGI2), a vasodilator and inhibitor of platelet aggregation, important for the vasodilatory response to nitric oxide (NO) [24]. The attenuation of these factors in patients with hypoalbuminemia may contribute to the increased risk of in-hospital and long-term mortality.

Whilst current evidence does not support the use of IV albumin treatment, an important modifiable target to increase albumin levels is nutritional intervention. Randomised control trials investigating nutritional intervention remain limited in patients with AIS [25,26], potentially driven by a lack of consensus on the benefits and optimal use of nutritional support in medical patients with acute and severe illness [27,28]. A small randomised controlled trial (RCT) recently showed improved albumin levels in patients with HF who received oral branched-chain amino acids (BCAAs) granules added to standard therapy for 28 days until discharge compared to nine controls [29]. Whether similar findings exist in stroke patients needs to be investigated. A RCT in 2020 demonstrates no rise in albumin after supplementation with whey protein in patients who have suffered a stroke [26]. A secondary post hoc analysis of the EFFORT RCT, concluded that in the short-term, a rise in serum albumin levels occurred regardless of nutritional support [27]. Further studies suggest that albumin levels may not depend on nutritional support or albumin treatment but rather the resolution of disease and inflammation [28]. Given the better outcomes associated with pre-discharge normalization of albumin levels [30] as well worse clinical outcomes shown in patients with hypoalbuminemia versus normal albumin levels, our findings highlight a pressing need for further RCTs to investigate interventions to increase albumin in patients who have suffered AIS. This may improve patient outcomes, particularly long-term mortality.

Our pooled analysis demonstrates an association of long-term mortality with low and low-normal albumin levels. Low albumin analysis revealed high heterogeneity, possibly driven by differences in effect size between the studies (35% increase in relative risk in our study vs. an almost two-fold increase in the study by Zhou et al. [4]). Differences may be attributed to a lack of adjustment for critical confounders, such as nutrition and thrombolysis, as well as variations in follow-up times. Importantly, the significantly smaller sample size comprising the low albumin quartile (<35 g/L) (7%) in Zhou et al.’s [4] study may contribute to larger variability and therefore greater heterogeneity. Importantly, our results highlight that clinical risk stratification strategies that only focus on hypoalbuminemia may insufficiently consider the increased risk of patients with low-normal albumin levels (35.0–39.9 g/L). This study demonstrates that albumin levels on admission after ischemic stroke may help stratify long-term risk of adverse stroke outcomes and identify patients who can benefit from stricter monitoring. This is supported by a recent large scale study showing albumin levels associated with risk of early cardiovascular events and death in patients with AIS [31].

The pooled effect size of high albumin levels was not significant for better functional outcomes or mortality. Prior studies, including a meta-analysis, suggest a better prognosis in patients with high albumin levels post-AIS [3,6,7,18]. However, these analyses assumed a linear relationship between albumin and outcomes, which our study and prior large-scale analyses found to not be the best-fitting model. Furthermore, patients with high albumin on admission are often haematologically concentrated, which may be fixed with fluids. The results may therefore be limited by single albumin measurements.

Our meta-analysis and primary analysis were powered by several strengths, including a large cohort of patients and long-term follow-up. Furthermore, our analyses adjusted for several important confounders, including, stroke severity, OCSP, WCC, and CRP. Additionally, we were able to relax the assumption that the relationship between albumin levels and outcomes was strictly linear by employing restricted cubic spline analyses. This allowed us to characterise the potential threshold effect occurring with albumin levels < 27–28 g/L.

We acknowledge some limitations. As a prospective population-based study, responder bias and residual confounding need to be considered. Albumin levels were measured once only, with prior studies demonstrating fluctuations after AIS [17,32]. Additionally, around 20% of the population had albumin measurements taken that varied in timing relative to admission. We could not adjust for stroke revascularisation therapy, due to too high missingness in the database. Creatinine levels may reflect malnutrition due to reduced muscle mass and be a confounding factor. Our data represent an older cohort, potentially affecting generalizability. Furthermore, studies have demonstrated a potential correlation between malnutrition with advanced age [33]. This may influence effect size in our study. Studies in the long-term mortality meta-analysis had high heterogeneity for low albumin (*I*^2^ > 50%).

## 5. Conclusions

In conclusion, hypoalbuminemia is a strong, independent prognostic marker of long-term mortality and worse functional outcomes. Our study demonstrates a non-linear relationship between albumin and clinical outcomes providing a more accurate representation of their relationship. Future research should include large-scale studies assessing this relationship, to quantify the evidence further, particularly for long-term mortality, where studies are currently limited. The therapeutic role of albumin and its application in risk stratification warrants further investigation.

## Figures and Tables

**Table 1:** Descriptive table.

	Total	Albumin QR1	Albumin QR2	Albumin QR3	Albumin QR4	*p* Value
N	9979 (0.00)	2808 (28.14)	2391 (23.96)	2510 (25.15)	2270 (22.75)	
Serum albumin, g/L, mean (SD)	36.69 (5.38)	30.09 (4.18)	36.08 (0.81)	38.95 (0.81)	43.01 (2.13)	<0.001
Age, years, mean (SD)	78.26 (11.23)	81.03 (10.33)	78.84 (10.98)	77.30 (11.07)	75.27 (11.84)	<0.001
Females	5219 (56.62)	1590 (56.62)	1255 (44.69)	1252 (44.59)	1122 (39.96)	<0.001
Comorbidities						
Hypertension	6138 (62.43)	1753 (62.43)	1485 (52.88)	1526 (54.34)	1374 (48.93)	0.419
Diabetes mellitus	1806 (20.80)	584 (20.80)	412 (14.67)	432 (15.38)	378 (13.46)	<0.001
Hyperlipidaemia	1370 (11.22)	315 (11.22)	350 (12.46)	374 (13.32)	331 (11.79)	<0.001
Coronary heart disease	2792 (31.37)	881 (31.37)	707 (25.18)	651 (23.18)	553 (19.69)	<0.001
Peripheral vascular disease	659 (8.87)	249 (8.87)	162 (5.77)	139 (4.95)	109 (3.88)	<0.001
Cerebrovascular disease	9867 (98.72)	2772 (98.72)	2367 (84.29)	2479 (88.28)	2249 (80.09)	0.566
Dementia	147 (2.35)	66 (2.35)	36 (1.28)	29 (1.03)	16 (0.57)	<0.001
Cancer	1590 (21.58)	606 (21.58)	373 (13.28)	370 (13.18)	241 (8.58)	<0.001
Renal disease	764 (12.71)	357 (12.71)	171 (6.09)	143 (5.09)	93 (3.31)	<0.001
Liver disease	151 (2.53)	71 (2.53)	37 (1.32)	29 (1.03)	14 (0.50)	<0.001
Pulmonary disease	1464 (18.41)	517 (18.41)	359 (12.78)	350 (12.46)	238 (8.48)	<0.001
COPD	826 (11.25)	316 (11.25)	204 (7.26)	201 (7.16)	105 (3.74)	<0.001
Pneumonia aspiration	845 (12.50)	351 (12.50)	182 (6.48)	169 (6.02)	143 (5.09)	<0.001
Pneumonia non-aspiration	1112 (18.27)	513 (18.27)	265 (9.44)	195 (6.94)	139 (4.95)	<0.001
OCSP types, *n* (%)						
Lacunar stroke (LACS)	2554 (21.87)	614 (21.87)	595 (21.19)	691 (24.61)	654 (23.29)	<0.001
Partial anterior circulation stroke (PACS)	3791 (37.96)	1066 (37.96)	960 (34.19)	962 (34.26)	803 (28.60)	0.010
Posterior circulation syndrome (POCS)	1651 (15.63)	439 (15.63)	392 (13.96)	415 (14.78)	405 (14.42)	0.213
Total anterior circulation stroke (TACS)	1983 (24.54)	689 (24.54)	444 (15.81)	442 (15.74)	408 (14.53)	<0.001
Pre-stroke mRs 0–2, *n* (%)	1292 (25.71)	451 (25.71)	326 (18.59)	296 (16.88)	219 (12.49)	<0.001
mRS at discharge, *n* (%)	1893 (57.88)	360 (57.88)	456 (73.31)	561 (90.19)	516 (82.96)	<0.001
Laboratory tests						
WBC, mmol/L, mean (SD)	8.80 (7.10–11.30)	9.30 (7.30–12.30)	8.40 (6.90–10.70)	8.50 (7.00–10.80)	9.00 (7.30–11.30)	<0.001
CRP, mmol/L, mean (SD)	11.00 (5.00–33.00)	30.00 (10.00–75.00)	9.00 (4.00–25.00)	8.00 (4.00–19.00)	8.00 (4.00–19.00)	<0.001
Medication						
Antiplatelet agents on discharge	5894 (49.00)	1376 (49.00)	1460 (51.99)	1632 (58.12)	1426 (50.78)	<0.001
Antiplatelet on admission	3657 (36.11)	1014 (36.11)	889 (31.66)	940 (33.48)	814 (28.99)	0.584
Anticoagulants on discharge	1200 (11.18)	314 (11.18)	315 (11.22)	303 (10.79)	268 (9.54)	0.173
Anticoagulant on admission	120 (1.35)	38 (1.35)	33 (1.18)	31 (1.10)	18 (0.64)	0.221
Outcomes						
Length of stay, *n* (IQR)	8.00 (4.00–17.29)	10.00 (4.21–22.00)	8.00 (3.43–17.00)	7.00 (3.00–15.22)	7.00 (4.00–16.00)	<0.001
NIHSS, *n* (IQR)	5.00 (2.00–13.00)	7.00 (3.00–14.00)	5.00 (2.00–13.00)	4.00 (2.00–12.00)	4.00 (2.00–10.00)	<0.001
Dead, *n* (%)	4645 (60.40)	1696 (60.40)	1035 (36.86)	953 (33.94)	961 (34.22)	<0.001
Poor outcomes (Rankin discharge ≥ 3)	5193(65.88)	1850(65.88)	1242(44.23)	1131(40.28)	970(34.54)	<0.001

## Data Availability

Data are contained within the article and Supplementary Materials.

## Supplementary Materials

The following supporting information can be downloaded at: https://www.mdpi.com/article/10.3390/nu16101486/s1, Supplemental Methods S1: Search strategy; Supplemental Methods S2: Statistical analysis for the meta-analysis Table S1: International Classification of Disease-tenth edition (ICD-10) codes; Table S2a–g: Missing data analysis tables for imputed variables; Table S3: Predictor Variables for Missing Data Analysis; Table S4: Summary table of studies assessing the relationship between serum albumin and poor functional outcome or mortality; Figure S1: Kaplan-Meier survival analysis of patients with AIS, categorized into four quartiles based on their albumin levels: QR1 (<35), QR2 (35–37), QR3 (38–41) and QR4 (>41); Figure S2: Spline models assessing the association between serum albumin and clinical outcomes without National Institute of Health Stroke Scale adjustment; Figure S3: Forest plots assessing the association between serum albumin and clinical outcomes; Figure S4: Forest plot assessing the association between serum albumin and clinical outcomes without National Institute of Health Stroke Scale adjustment; Figure S5: Meta-analysis of the association of categorical serum albumin levels with poor functional outcomes; Figure S6: Sensitivity analyses of poor functional outcomes by omitting Thuemmler et al., due variation in follow up; Figure S7: Sensitivity analyses of poor functional outcomes by omitting Thuemmler et al., due variation in follow up. Reference [34] is cited in the Supplementary Materials.
