# Supplementary material for: Serum Albumin and Post-Stroke Outcomes: Analysis of UK Regional Registry Data, Systematic Review, and Meta-Analysis
Source: Nutrients. 2024 May 14;16(10):1486. doi: 10.3390/nu16101486 (PMC11124370; doi:10.3390/nu16101486)
Supplement: Supplementary file 1 [file nutrients-16-01486-s001.zip › nutrients-2951989-supplementary.pdf]

Detailed Methods

Supplementary Methods S1. Search strategy.

Embase

Albumin ((exp albumin/) OR (exp hypoalbuminemia/)) **And Stroke** ((exp cerebrovascular accident/) OR (stroke*.tw.) OR (“cerebral isch?mia".tw.) OR (TIA.tw.) OR ("acute isch?mic infarct".tw.)) **AND Outcomes ((**outcome.tw.) OR (mortality.tw.) OR (“length of stay".tw.) OR ("modified rankin scale".tw.) OR (“mRS.tw.”) OR (functional.tw.) OR (exp prognosis/) OR (dependency.tw.))

MEDLINE(R)

Albumin ((exp Albumins/) OR (exp Serum Albumin/) OR (exp Hypoalbuminemia)) **And Stroke ((**exp Stroke/) OR **(**exp Ischemic Stroke/) OR **(**stroke*.tw.) OR ("cerebral isch*mia".tw.) OR **(**"acute isch*mic infarct".tw.) OR (TIA.tw.) OR ("ischaemic stroke".tw.) OR ("cerebrovascular accident".tw.)) **AND Outcomes** (outcome.tw.) OR (mortality.tw.) OR ("length of stay".tw.) OR ("modified rankin scale".tw.) OR (mRS.tw.) OR (functional.tw.) OR (exp Prognosis/) OR (dependency.tw.)

Scopus

Albumin TITLE-ABS-KEY (albumin OR "serum albumin" OR hypoalbuminemia) **And Stroke** TITLE-ABS-KEY (stroke* OR "ischemic stroke" OR "ischaemic stroke" OR "cerebral ischaemia" OR "cerebral ischemia" OR tia OR "cerebrovascular accident”) **AND Outcomes** TITLE-ABS-KEY (outcome OR mortality OR "length of stay" OR "modified rankin scale" OR mrs OR functional OR prognosis OR dependency))

Supplementary Methods S2. Statistical analysis for the meta-analysis.

Whilst the studies presented in the previous meta-analysis by Zhou [1] et al, were considered, our analysis was based on the effects size of dose stratified albumin opposed to albumin as a continuous variable and therefore multiple studies were excluded due to heterogeneity. Four[2–5] out of six studies[1–5] were excluded from the long-term mortality analyses. Gariballa [2] *et al*, reported a HR of 0.91(95% CI 0.84 - 0.99) for every +1g/L increment in albumin. The study provided no information on the distribution of albumin across quartiles and therefore these results could not be converted for HR comparing albumin quartiles to be included in the meta-analysis. Similarly, Carter Am *et al*[3] reported a HR of 0.65 (95% CI 0.4 - 0.96) for albumin levels >43g/L vs 38g/L. This study required extraction of effect estimates from a Kaplan Maier curve to determine effect sizes of albumin as a categorical variable which was determined to not meet the required level of rigor for our analysis. Idicula [4] TT *et al* reports an OR of 0.88 (95% CI 0.83 - 0.93) per +1g/L and Lazaro[5] *et al* reports an OR of 2.00 (95% CI 1.12 - 2.3) per -1g/L. As both studies reported odds ratios instead of hazard ratios, these could not be included in a meta-analysis of studies reporting HR[6].

For functional outcomes Yang *et al.*[7] was excluded from this outcome. This was due to the population only including patients treated with IVT. In comparison to a reference group of <39.3g/L, albumin groups 39.3g/L - 42.0g/L, 42.1g/L - 43.5g/L, >43.5g/L showed no association with functional outcomes. Four studies, including our own were included in the assessment of poor functional outcome. Dziedzic [8] *et al* and Babu [9] *et al* had to be converted from a continuous scale (g/L and g/dL) into categorical variables. The OR per 1g/L serum albumin increases was converted to per1g/L serum albumin decrease by taking the inverse. Due to the assumption of linearity of the reported results, it was possible to calculate an

estimated OR for the desired quartiles. The third quartile group (40-44.9g/L) was used as the reference group, based on the medium albumin value of our population and previous studies.

Table S1. International Classification of Disease-tenth edition (ICD-10) codes.

Pulmonary disease	J40-J47, J60-J67
Atrial fibrillation	I48
Previous cerebrovascular disease	I60-I66, I670-I672, I674-I679, G450-G452, G454, G458-G46
Coronary heart disease	120-125
Congestive heart failure	I50
Chronic kidney disease	N18
Dementia	F00-F05
Diabetes mellitus	E10-E14
Hyperlipidaemia	E78
Hypertension	I10-I15
Liver disease	K70-K77
Malignancy	C00-C97
Peptic ulcer disease	K25-K28
Peripheral vascular disease	i71, I790, R02, Z958, Z959
Rheumatoid arthritis/connective tissue disease	M32, M34, M332, M053, M058, M059, M060, M063, M069, M050, M052, M051, M353

Supplementary Tables S2a – S2g: Missing data analysis tables for imputed variables

Table S2a: Missing data analysis for Pre-stroke modified Rankin Scale.

Pre-stroke modified Rankin Scale	Total	Non-missing	Missing	P value
N	9979	9445	534	
Age, years, mean (SD)	78.26 (11.23)	78.11 (11.25)	80.88 (10.49)	<0.001
Women	5219 (52.30)	4926 (52.15)	293 (54.87)	0.222
Comorbidities				
Hypertension	6138 (61.51)	5866 (62.11)	272 (50.94)	<0.001
Diabetes	1806 (18.10)	1712 (18.13)	94 (17.60)	0.760
Hyperlipidemia	1370 (13.73)	1332 (14.10)	38 (7.12)	<0.001
Chronic Heart Disease	2792 (27.98)	2647 (28.03)	145 (27.15)	0.662
Atrial Fibrillation and Atrial Flutter	3264 (32.71)	3089 (32.71)	175 (32.77)	0.975
Peripheral Vascular Disease	659 (6.60)	619 (6.55)	40 (7.49)	0.396
Peptic Ulcer Disease	437 (4.38)	403 (4.27)	34 (6.37)	0.021
Connective Tissue Disease	525 (5.26)	501 (5.30)	24 (4.49)	0.415
Cerebral Vascular Disease	9867 (98.88)	9347 (98.96)	520 (97.38)	<0.001
Dementia	147 (1.47)	139 (1.47)	8 (1.50)	0.961
Cancer	1590 (15.93)	1486 (15.73)	104 (19.48)	0.022
Renal Diseases	764 (7.66)	715 (7.57)	49 (9.18)	0.175
Liver Disease	151 (1.51)	143 (1.51)	8 (1.50)	0.977
Chronic Pulmonary Disease	1464 (14.67)	1377 (14.58)	87 (16.29)	0.276
COPD	826 (8.28)	774 (8.19)	52 (9.74)	0.208
Pneumonia (Aspiration)	845 (8.47)	784 (8.30)	61 (11.42)	0.012
Pneumonia (Non-Aspiration)	1112 (11.14)	1027 (10.87)	85 (15.92)	<0.001
Mediation				
Antiplatelet agents on discharge	5894 (59.06)	5759 (60.97)	135 (25.28)	<0.001
Anticoagulants on discharge	1200 (12.03)	1179 (12.48)	21 (3.93)	<0.001
Antiplatelet agents on admission	3657 (36.65)	3526 (37.33)	131 (24.53)	<0.001
Anticoagulant agents on admission	120 (1.20)	120 (1.27)	0 (0.00)	0.009
Outcomes				
Died	4645 (46.55)	4257 (45.07)	388 (72.66)	<0.001
Length of stay	8.00 (4.00-17.29)	8.00 (4.00-17.00)	13.00 (5.00-26.00)	<0.001

Table S2b: Missing data analysis for Rankin Discharge.

Rankin Discharge	Total	Non-missing	Missing	P value
N	9979	6967	3012	
Age, years, mean, (SD)	78.26 (11.23)	78.66 (11.40)	77.34 (10.78)	<0.001
Females	5219 (52.30)	3624 (52.02)	1595 (52.95)	0.389

Comorbidities				
Hypertension	6138 (61.51)	4486 (64.39)	1652 (54.85)	<0.001
Diabetes	1806 (18.10)	1359 (19.51)	447 (14.84)	<0.001
Hyperlipidemia	1370 (13.73)	1119 (16.06)	251 (8.33)	<0.001
Chronic Heart Disease	2792 (27.98)	2048 (29.40)	744 (24.70)	<0.001
Atrial Fibrillation and Atrial Flutter	3264 (32.71)	2425 (34.81)	839 (27.86)	<0.001
Peripheral Vascular Disease	659 (6.60)	486 (6.98)	173 (5.74)	0.023
Peptic Ulcer Disease	437 (4.38)	299 (4.29)	138 (4.58)	0.516
Connective Tissue Disease	525 (5.26)	407 (5.84)	118 (3.92)	<0.001
Cerebral Vascular Disease	9867 (98.88)	6897 (99.00)	2970 (98.61)	0.090
Dementia	147 (1.47)	127 (1.82)	20 (0.66)	<0.001
Cancer	1590 (15.93)	1188 (17.05)	402 (13.35)	<0.001
Renal Diseases	764 (7.66)	612 (8.78)	152 (5.05)	<0.001
Liver Disease	151 (1.51)	122 (1.75)	29 (0.96)	0.003
Chronic Pulmonary Disease	1464 (14.67)	1121 (16.09)	343 (11.39)	<0.001
COPD	826 (8.28)	644 (9.24)	182 (6.04)	<0.001
Pneumonia (Aspiration)	845 (8.47)	746 (10.71)	99 (3.29)	<0.001
Pneumonia (Non-Aspiration)	1112 (11.14)	941 (13.51)	171 (5.68)	<0.001
Treatment				
Antiplatelet agents on discharge	5894 (59.06)	3935 (56.48)	1959 (65.04)	<0.001
Anticoagulants on discharge	1200 (12.03)	856 (12.29)	344 (11.42)	0.222
Antiplatelet agents on admission	3657 (36.65)	2493 (35.78)	1164 (38.65)	0.006
Anticoagulant agents on admission	120 (1.20)	120 (1.72)	0 (0.00)	<0.001
Outcomes				
Died	4645 (46.55)	2856 (40.99)	1789 (59.40)	<0.001
Length of stay	8.00 (4.00-17.29)	6.00 (3.00-15.00)	12.00 (6.00-23.00)	<0.001

Table S2c: Missing data analysis for NIHSS.

NIHSS	Total	Non-missing	Missing	P value
N	9979	1039	8940	
Age, years, mean (SD)	78.26 (11.23)	77.08 (12.01)	78.40 (11.13)	<0.001
Women	5219 (52.30)	506 (48.70)	4713 (52.72)	0.014
Comorbidities				
Hypertension	6138 (61.51)	678 (65.26)	5460 (61.07)	0.009
Diabetes	1806 (18.10)	201 (19.35)	1605 (17.95)	0.270
Hyperlipidemia	1370 (13.73)	206 (19.83)	1164 (13.02)	<0.001
Chronic Heart Disease	2792 (27.98)	271 (26.08)	2521 (28.20)	0.150
Atrial Fibrillation and Atrial Flutter	3264 (32.71)	312 (30.03)	2952 (33.02)	0.052

Peripheral Vascular Disease	659 (6.60)	74 (7.12)	585 (6.54)	0.477
Peptic Ulcer Disease	437 (4.38)	54 (5.20)	383 (4.28)	0.173
Connective Tissue Disease	525 (5.26)	65 (6.26)	460 (5.15)	0.129
Cerebral Vascular Disease	9867 (98.88)	1038 (99.90)	8829 (98.76)	<0.001
Dementia	147 (1.47)	19 (1.83)	128 (1.43)	0.315
Cancer	1590 (15.93)	194 (18.67)	1396 (15.62)	0.011
Renal Diseases	764 (7.66)	108 (10.39)	656 (7.34)	<0.001
Liver Disease	151 (1.51)	19 (1.83)	132 (1.48)	0.379
Chronic Pulmonary Disease	1464 (14.67)	200 (19.25)	1264 (14.14)	<0.001
COPD	826 (8.28)	110 (10.59)	716 (8.01)	0.004
Pneumonia (Aspiration)	845 (8.47)	44 (4.23)	801 (8.96)	<0.001
Pneumonia (Non-Aspiration)	1112 (11.14)	129 (12.42)	983 (11.00)	0.168
Medication				
Antiplatelet agents on discharge	5894 (59.06)	746 (71.80)	5148 (57.58)	<0.001
Anticoagulants on discharge	1200 (12.03)	210 (20.21)	990 (11.07)	<0.001
Antiplatelet agents on admission	3657 (36.65)	104 (10.01)	3553 (39.74)	<0.001
Anticoagulant agents on admission	120 (1.20)	103 (9.91)	17 (0.19)	<0.001
Outcome				
Died	4645 (46.55)	190 (18.29)	4455 (49.83)	<0.001
Length of stay	8.00 (4.00-17.29)	4.81 (2.22-12.76)	9.00 (4.00-18.00)	<0.001

Table S2d: Missing data analysis for OCSP classification.

Bamford	Total	Non-missing	Missing	P value
n	9979	9005	974	
Age, years, mean, (SD)	78.26 (11.23)	78.23 (11.21)	78.50 (11.44)	0.477
Women	5219 (52.30)	4727 (52.49)	492 (50.51)	0.240
Comorbidities				
Hypertension	6138 (61.51)	5558 (61.72)	580 (59.55)	0.185
Diabetes	1806 (18.10)	1630 (18.10)	176 (18.07)	0.981
Hyperlipidemia	1370 (13.73)	1236 (13.73)	134 (13.76)	0.978
Chronic Heart Disease	2792 (27.98)	2532 (28.12)	260 (26.69)	0.347
Atrial Fibrillation and Atrial Flutter	3264 (32.71)	2933 (32.57)	331 (33.98)	0.372
Peripheral Vascular Disease	659 (6.60)	603 (6.70)	56 (5.75)	0.258
Peptic Ulcer Disease	437 (4.38)	390 (4.33)	47 (4.83)	0.474
Connective Tissue Disease	525 (5.26)	481 (5.34)	44 (4.52)	0.274
Cerebral Vascular Disease	9867 (98.88)	8905 (98.89)	962 (98.77)	0.732
Dementia	147 (1.47)	129 (1.43)	18 (1.85)	0.307
Cancer	1590 (15.93)	1404 (15.59)	186 (19.10)	0.005
Renal Diseases	764 (7.66)	666 (7.40)	98 (10.06)	0.003
Liver Disease	151 (1.51)	128 (1.42)	23 (2.36)	0.022

Chronic Pulmonary Disease	1464 (14.67)	1316 (14.61)	148 (15.20)	0.626
COPD	826 (8.28)	738 (8.20)	88 (9.03)	0.366
Pneumonia (Aspiration)	845 (8.47)	740 (8.22)	105 (10.78)	0.006
Pneumonia (Non-Aspiration)	1112 (11.14)	980 (10.88)	132 (13.55)	0.012
Treatment				
Antiplatelet agents on discharge	5894 (59.06)	5418 (60.17)	476 (48.87)	<0.001
Anticoagulants on discharge	1200 (12.03)	1104 (12.26)	96 (9.86)	0.028
Antiplatelet agents on admission	3657 (36.65)	3298 (36.62)	359 (36.86)	0.885
Anticoagulant agents on admission	120 (1.20)	114 (1.27)	6 (0.62)	0.077
Outcomes				
Died	4645 (46.55)	4220 (46.86)	425 (43.63)	0.055
Length of Stay	8.00 (4.00-17.29)	8.00 (4.00-18.00)	6.02 (3.00-16.00)	<0.001

Table S2e: Missing data analysis for Albumin at admission.

Albumin	Total	Non-missing	Missing	P value
n	9979	9592	387	
Age, years, mean, (SD)	78.26 (11.23)	78.30 (11.22)	77.15 (11.43)	0.048
Females	5219 (52.30)	5038 (52.52)	181 (46.77)	0.026
Comorbidities				
Hypertension	6138 (61.51)	5920 (61.72)	218 (56.33)	0.033
Diabetes	1806 (18.10)	1734 (18.08)	72 (18.60)	0.792
Hyperlipidemia	1370 (13.73)	1316 (13.72)	54 (13.95)	0.896
Chronic Heart Disease	2792 (27.98)	2711 (28.26)	81 (20.93)	0.002
Atrial Fibrillation and Atrial Flutter	3264 (32.71)	3172 (33.07)	92 (23.77)	<0.001
Peripheral Vascular Disease	659 (6.60)	636 (6.63)	23 (5.94)	0.593
Peptic Ulcer Disease	437 (4.38)	422 (4.40)	15 (3.88)	0.622
Connective Tissue Disease	525 (5.26)	513 (5.35)	12 (3.10)	0.052
Cerebral Vascular Disease	9867 (98.88)	9501 (99.05)	366 (94.57)	<0.001
Dementia	147 (1.47)	142 (1.48)	5 (1.29)	0.763
Cancer	1590 (15.93)	1530 (15.95)	60 (15.50)	0.814
Renal Diseases	764 (7.66)	741 (7.73)	23 (5.94)	0.196
Liver Disease	151 (1.51)	148 (1.54)	3 (0.78)	0.225
Chronic Pulmonary Disease	1464 (14.67)	1407 (14.67)	57 (14.73)	0.974
COPD	826 (8.28)	798 (8.32)	28 (7.24)	0.448
Pneumonia (Aspiration)	845 (8.47)	825 (8.60)	20 (5.17)	0.017
Pneumonia (Non-Aspiration)	1112 (11.14)	1082 (11.28)	30 (7.75)	0.031
Treatment				
Antiplatelet agents on discharge	5894 (59.06)	5706 (59.49)	188 (48.58)	<0.001

Anticoagulants on discharge	1200 (12.03)	1174 (12.24)	26 (6.72)	0.001
Antiplatelet agents on admission	3657 (36.65)	3530 (36.80)	127 (32.82)	0.111
Anticoagulant agents on admission	120 (1.20)	118 (1.23)	2 (0.52)	0.207
Outcomes				
Died	4645 (46.55)	4491 (46.82)	154 (39.79)	0.007
Length of stay	8.00 (4.00-17.29)	8.00 (4.00-18.00)	5.00 (2.00-11.00)	<0.001

Table S2f: Missing data analysis for WBC at admission.

WBC	Total	Non-missing	Missing	P value
n	9979	9773	206	
Age, years, mean, (SD)	78.26 (11.23)	78.26 (11.23)	77.96 (11.05)	0.703
Females	5219 (52.30)	5120 (52.39)	99 (48.06)	0.218
Comorbidities				
Hypertension	6138 (61.51)	6027 (61.67)	111 (53.88)	0.023
Diabetes	1806 (18.10)	1760 (18.01)	46 (22.33)	0.111
Hyperlipidemia	1370 (13.73)	1339 (13.70)	31 (15.05)	0.578
Chronic Heart Disease	2792 (27.98)	2757 (28.21)	35 (16.99)	<0.001
Atrial Fibrillation and Atrial Flutter	3264 (32.71)	3210 (32.85)	54 (26.21)	0.045
Peripheral Vascular Disease	659 (6.60)	648 (6.63)	11 (5.34)	0.460
Peptic Ulcer Disease	437 (4.38)	430 (4.40)	7 (3.40)	0.487
Connective Tissue Disease	525 (5.26)	520 (5.32)	5 (2.43)	0.066
Cerebral Vascular Disease	9867 (98.88)	9680 (99.05)	187 (90.78)	<0.001
Dementia	147 (1.47)	143 (1.46)	4 (1.94)	0.573
Cancer	1590 (15.93)	1555 (15.91)	35 (16.99)	0.675
Renal Diseases	764 (7.66)	754 (7.72)	10 (4.85)	0.126
Liver Disease	151 (1.51)	149 (1.52)	2 (0.97)	0.519
Chronic Pulmonary Disease	1464 (14.67)	1437 (14.70)	27 (13.11)	0.521
COPD	826 (8.28)	815 (8.34)	11 (5.34)	0.122
Pneumonia (Aspiration)	845 (8.47)	835 (8.54)	10 (4.85)	0.060
Pneumonia (Non-Aspiration)	1112 (11.14)	1092 (11.17)	20 (9.71)	0.508
Treatment				
Antiplatelet agents on discharge	5894 (59.06)	5808 (59.43)	86 (41.75)	<0.001
Anticoagulants on discharge	1200 (12.03)	1189 (12.17)	11 (5.34)	0.003
Antiplatelet agents on admission	3657 (36.65)	3595 (36.79)	62 (30.10)	0.049
Anticoagulant agents on admission	120 (1.20)	119 (1.22)	1 (0.49)	0.340
Outcomes				
Died	4645 (46.55)	4572 (46.78)	73 (35.44)	0.001

Length of stay	8.00 (4.00-17.29)	8.00 (4.00-18.00)	5.00 (2.00-12.00)	<0.001

Table S2g: Missing data analysis for CRP at admission.

CRP	Total	Non-missing	Missing	P value
n	9979	8298	1681	
Age, years, mean, (SD)	78.26 (11.23)	78.87 (10.96)	75.22 (12.03)	<0.001
Females	5219 (52.30)	4392 (52.93)	827 (49.20)	0.005
Comorbidities				
Hypertension	6138 (61.51)	5207 (62.75)	931 (55.38)	<0.001
Diabetes	1806 (18.10)	1558 (18.78)	248 (14.75)	<0.001
Hyperlipidemia	1370 (13.73)	1123 (13.53)	247 (14.69)	0.208
Chronic Heart Disease	2792 (27.98)	2418 (29.14)	374 (22.25)	<0.001
Atrial Fibrillation and Atrial Flutter	3264 (32.71)	2908 (35.04)	356 (21.18)	<0.001
Peripheral Vascular Disease	659 (6.60)	593 (7.15)	66 (3.93)	<0.001
Peptic Ulcer Disease	437 (4.38)	394 (4.75)	43 (2.56)	<0.001
Connective Tissue Disease	525 (5.26)	476 (5.74)	49 (2.91)	<0.001
Cerebral Vascular Disease	9867 (98.88)	8220 (99.06)	1647 (97.98)	<0.001
Dementia	147 (1.47)	132 (1.59)	15 (0.89)	0.030
Cancer	1590 (15.93)	1374 (16.56)	216 (12.85)	<0.001
Renal Diseases	764 (7.66)	702 (8.46)	62 (3.69)	<0.001
Liver Disease	151 (1.51)	140 (1.69)	11 (0.65)	0.002
Chronic Pulmonary Disease	1464 (14.67)	1267 (15.27)	197 (11.72)	<0.001
COPD	826 (8.28)	741 (8.93)	85 (5.06)	<0.001
Pneumonia (Aspiration)	845 (8.47)	801 (9.65)	44 (2.62)	<0.001
Pneumonia (Non-Aspiration)	1112 (11.14)	1027 (12.38)	85 (5.06)	<0.001
Treatment				
Antiplatelet agents on discharge	5894 (59.06)	4845 (58.39)	1049 (62.40)	0.002
Anticoagulants on discharge	1200 (12.03)	1051 (12.67)	149 (8.86)	<0.001
Antiplatelet agents on admission	3657 (36.65)	3078 (37.09)	579 (34.44)	0.040
Anticoagulant agents on admission	120 (1.20)	111 (1.34)	9 (0.54)	0.006
Outcome				
Died	4645 (46.55)	4078 (49.14)	567 (33.73)	<0.001
LoS	8.00 (4.00-17.29)	9.00 (4.00-20.00)	5.00 (2.02-9.00)	<0.001

Table S3. Predictor Variables for Missing Data Analysis

Age	Liver disease
Women	Cancer
Pulmonary Disease	Metastatic Cancer
COPD	Peptic Ulcer Disease
Atrial Fibrillation and Flutter	Transient Ischaemic Attack
Cerebral vascular disease	Pneumonia (Including aspiration)
Chronic heart disease	Acute myocardial infarction
Congestive heart failure	Peripheral vascular disease
Renal Disease	Connective tissue disease
Dementia	Rheumatoid arthritis
Diabetes	Antiplatelet medication on discharge
Hyperlipidaemia	Anticoagulation medication on discharge
Hypertension	Length of stay

Table S4. Summary table of studies assessing the relationship between serum albumin and poor functional outcome or mortality.

	Country	No. of subjects	Participants	Age, years	Men, %	Follow-up time	Albumin comparison	Study outcome	Adjustment	Effect Estimate	Categorical Estimate	ROB
Gariballa SE et al, 1998[2]	UK	225	Acute Ischemic Stroke	77.6 ∓9.4	96(42.7)	3 months	Per +1 g/L	Mortality	Age, sex, mRS, previous illnesses, drugs, smoking	HR:0.91(0.84-0.99)	-	High
Dziedzic T et al, 2004[8]	Poland	759	Acute Ischemic Stroke	68.3 ∓12	372(49.0)	3 months	Per +1 g/L	Poor outcome (mRS 4-6)	Age, sex, atrial fibrillation, ischaemic heart disease, smoking, SSS score on admission, infarct size, TC	OR:0.96(0.93-0.99)	QR 1:3 OR:1.67(1.13-2.47) QRT 2:3 OR: 1.15(1.04-1.29) QR 4:3 OR: 0.60(0.40-0.97)	High
Carter[3] AM et al, 2007	UK	545	Acute Ischemic Stroke	-	274(50.3)	7.4 years(median)	>43g/L vs <38g/L	Mortality	Age, stroke subtype, previous stroke/TIA, atrial fibrillation, creatinine, haemoglobulin, finbrinogen, FVIII, FXIIIA, beta-TG, vWF, tPA	HR:0.65(0.44-0.96)	-	High
Idicula TT[4] et al, 2009	Norway	444	Acute Ischemic Stroke	70.3 ∓14.4	250(56.3)	2 years	Per +1 g/L	Mortality	Age, sex and NIHSS score on admission	OR:0.88(0.83-0.93)	-	High
Alcazar Lazaro V[5] et al, 2013	Spain	260	Acute Ischemic Stroke	-	127(48.8)	5 years	Per -1 g/L	Mortality	Age, BMI, cardiopathy, atrial fibrillation, urea, calcemia, total proteins, cholesterol, glycaemia, embolic mechanism, coma, DBP, Canadian scale score on admission	OR:2.00(1.12-3)	-	High

Babu MS[9] et al, 2013	India	560	Acute Ischemic Stroke	-	401(71.6)	3 months	Per -1 g/L	Poor outcome (mRS 4-6)	Age, sex, smoking, diabetes, hypertension, alcoholism, TC, HDL-C, LDL-C and TG	OR: 1.972(1.103-4.001)	Q1:3 OR: 2.33(1.13-5.68) Q2:3 OR:1.27(1.05-1.63) Q4:3 OR:0.713(0.50-0.96)	Some concern
Zhou HY[1] et al, 2020	China	13618	Acute Ischemic Stroke or transient ischemic attack	62.17 ∓11.26	9276 (68.12)	1 year	Q1:3 [<35 vs 40-44.9] Q2:3 [35-39.9 vs 40-44.9] Q4:3[>45 vs 40-44.9]	Poor outcome	Age, sex, BMI, medical history (hypertension, diabetes mellitus, stroke or TIA, coronary heart disease and atrial fibrillation/flutter), diagnosis type, TOAST type, NIHSS score on admission, Pre-stroke mRS 0-2 on admission, intracranial arterial stenosis, extracranial arterial stenosis, intravenous thrombolysis, inpatient medication (antihypertensive agents, anticoagulation agents) , TG, TC, LDL, HDL, ALT, eGFR, hs-CRP)	Q1:3 OR:1.37(1.12-1.67) Q2:3 0.99(0.88-1.13) Q3:4 0.95(0.79-1.16)	Q1:3 OR:1.37(1.12-1.67) Q2:3 0.99(0.88-1.13) Q3:4 0.95(0.79-1.16)	Some concern
								Mortality		Q1:3 HR:1.93(1.45 to 2.57) Q2:3 HR:1.12 (0.89-1.41) Q3:4 HR:1.14(1.03-1.26)	Q1:3 HR:1.93(1.45 to 2.57) Q2:3 HR:1.12 (0.89-1.41) Q3:4 HR:1.14(1.03-1.26)	Some concern
Yang et al[7], 2021	China	142	Acute Ischemic Stroke with EVT	-	196(63.01)	3 months	Q1 [<39.93] Q2 [39.3-42.0] Q3 [42.1-43.5]	Poor functional outcome (mRS = 3-6)	Age, sex, current smoking, and NIHSS at admission)	Q2:1 OR: 0.541(0.137-2.136) Q3:1 OR: 0.609(0.148-2.509)	Q1 OR: - Q2 OR: 0.541(0.137-2.136)	High

							Q4 [>43.5]			Q4:1 OR: 0.702(0.194-2.534)	Q3 OR: 0.609(0.148-2.509)<br>Q4 OR: 0.702(0.194-2.534)	
Thuemmler et al, 2023	UK	9979	Acute Ischemic stroke	78.26 ∓11.23	4290(43.38)	In-hospital	Q1:3 [<35 vs 40-44]<br>Q2:3 [35-39.9 vs 40-44]<br>Q4:3[42-47 vs 40-44]	Poor outcome	Age sex, OCSP, Pre-stroke (mRS 0-2) on admission, NIHSS score on admission, medical history (hypertension, diabetes, coronary heart disease, atrial fibrillation, chronic kidney disease, peripheral vascular disease, hyperlipidaemia, liver disease, chronic pulmonary disease, COPD, chronic heart failure, peptic ulcer disease, cerebrovascular disease, cancer, dementia, connective tissue disease) inpatient medication (antiplatelets at discharge, anti-coagulation therapy at discharge) CRP, WBC	Q1:3 RR: 1.51(1.29-1.76)<br>Q1:3 RR: 1.18(1.02-1.37)<br>Q4:3 RR: 1.18(1.02-1.37)	Q1:3 RR: 1.51(1.29-1.76)<br>Q1:3 RR: 1.18(1.02-1.37)<br>Q4:3 RR: 1.18(1.02-1.37)	Some concern
						5.5 years		Mortality		QR1 HR:1.35(1.22-1.49)<br>QR2 HR: 1.10(1.00-1.21)<br>QR3 HR:1.14(1.03-1.26)	QR1 HR:1.35(1.22-1.49)<br>QR2 HR: 1.10(1.00-1.21)<br>QR3 HR:1.14(1.03-1.26)	Some concern

BMI, body mass index; TOAST, the Trial of Org 10172 in Acute Stroke Treatment; NIHSS, National Institute of Health Stroke Scale; mRS, modified Rankin Score; OCSP, Oxford Community Stroke Project; ROB, Risk of Bias; TC, total cholesterol; HD:, high-denisty lipoprotein, low-density

lipoprotein; TG, triglyceride; eGFR, estimated glomerular filtration rate; ALT, alanine aminotransferase; hs-CRP, high-sensitivity C-reactive protein; DBP, diastolic blood pressure; vWF, von Willebrand factor; tPA, tissue-type plasminogen activator; OR, odds ratio; HR, hazard ratio

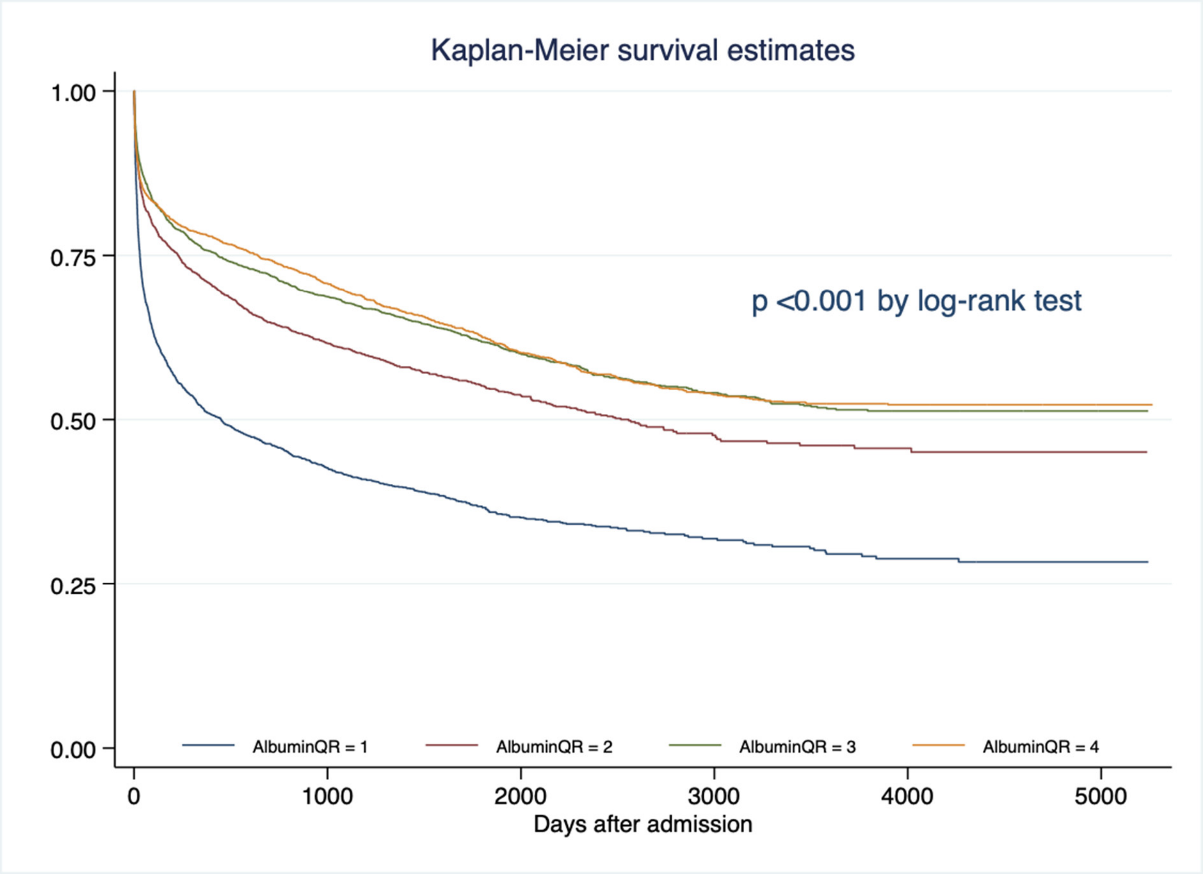

Figure S1. Kaplan-Meier survival analysis of patients with AIS, categorized into four quartiles based on their albumin levels: QR1 (<35), QR2 (35-37), QR3 (38-41) and QR4 (>41). Patients with low albumin levels were more likely to be dead at long-term follow up (p<0.001 by log-rank test).

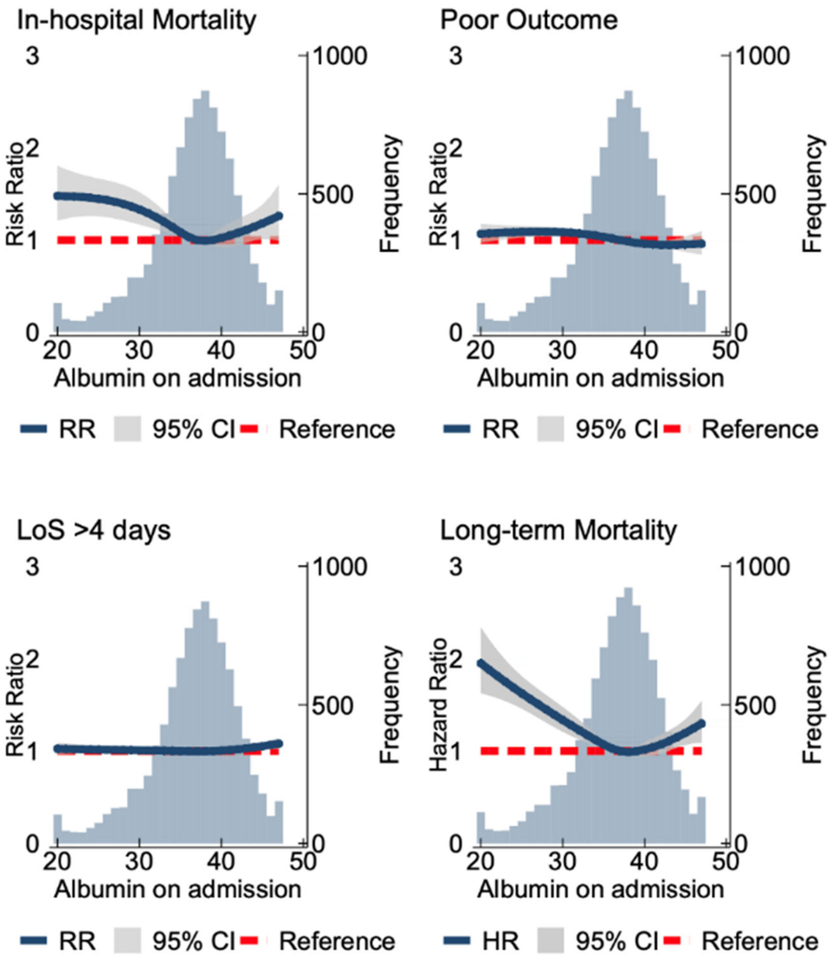

Figure S2. Spline models assessing the association between serum albumin and clinical outcomes without National Institute of Health Stroke Scale adjustment. The association with serum albumin level and in-hospital mortality, poor functional outcomes (modified Rankin Scale(mRS) score of 3-6) and increased length of stay (>4days). The association of serum albumin and long-term mortality at 5.5 year follow up. The blue line indicates the RR/HR, and the grey area indicates the confidence interval. The red line demonstrates the null effect line. The long-term mortality models were fitted by a Cox regression with adjustments made for age, sex comorbidities(chronic pulmonary disease, atrial fibrillation, cerebrovascular disease, congestive heart disease, heart failure, renal disease, chronic obstructive pulmonary

disease, dementia, diabetes, hypertension, hyperlipidaemia, liver disease, cancer, peptic ulcer disease, peripheral disease and connective tissue disease, pneumonia (aspiration and non-aspiration), anti-platelet and anti-coagulation medication on discharge/admission, OSCP scale, pre-stroke mRS score, serum white blood count, C-reactive protein.

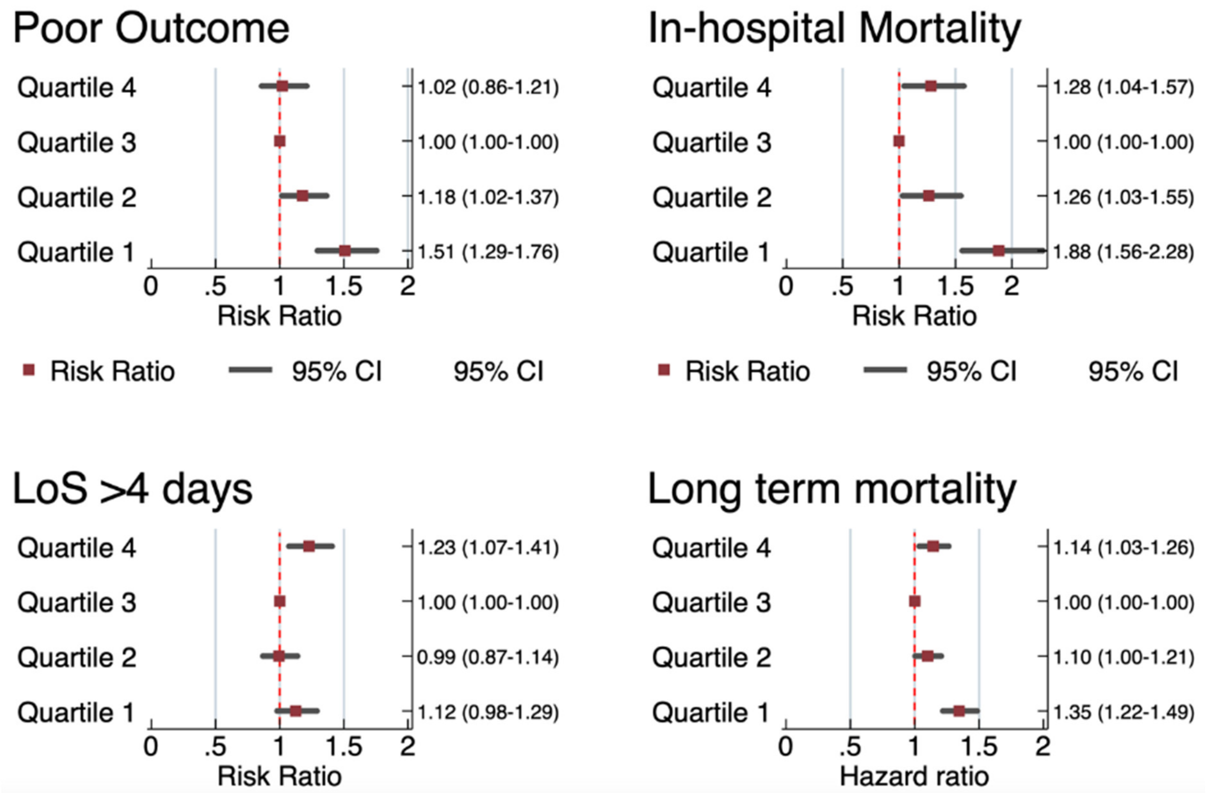

Figure S3. Forest plots assessing the association between serum albumin and clinical outcomes. The association with serum albumin level and in-hospital mortality, poor functional outcomes (modified Rankin Scale(mRS) score of 3-6) and increased length of stay (>4days). The association of serum albumin and long-term mortality at 5.5 year follow up. The blue line indicates the RR/HR, and the grey area indicates the confidence interval. The red line demonstrates the null effect line. The long-term mortality models were fitted by a Cox regression with adjustments made for age, sex comorbidities(chronic pulmonary disease, atrial fibrillation, cerebrovascular disease, congestive heart disease, heart failure, renal disease, COPD, dementia, diabetes, hypertension, hyperlipidaemia, liver disease, cancer, peptic ulcer disease, peripheral disease and connective tissue disease, pneumonia (aspiration and non-aspiration), anti-platelet and anti-coagulation medication on discharge/admission, National Institute of Health Stroke Scale, OSCP scale, pre-stroke mRS score, serum white blood count and C-reactive protein.

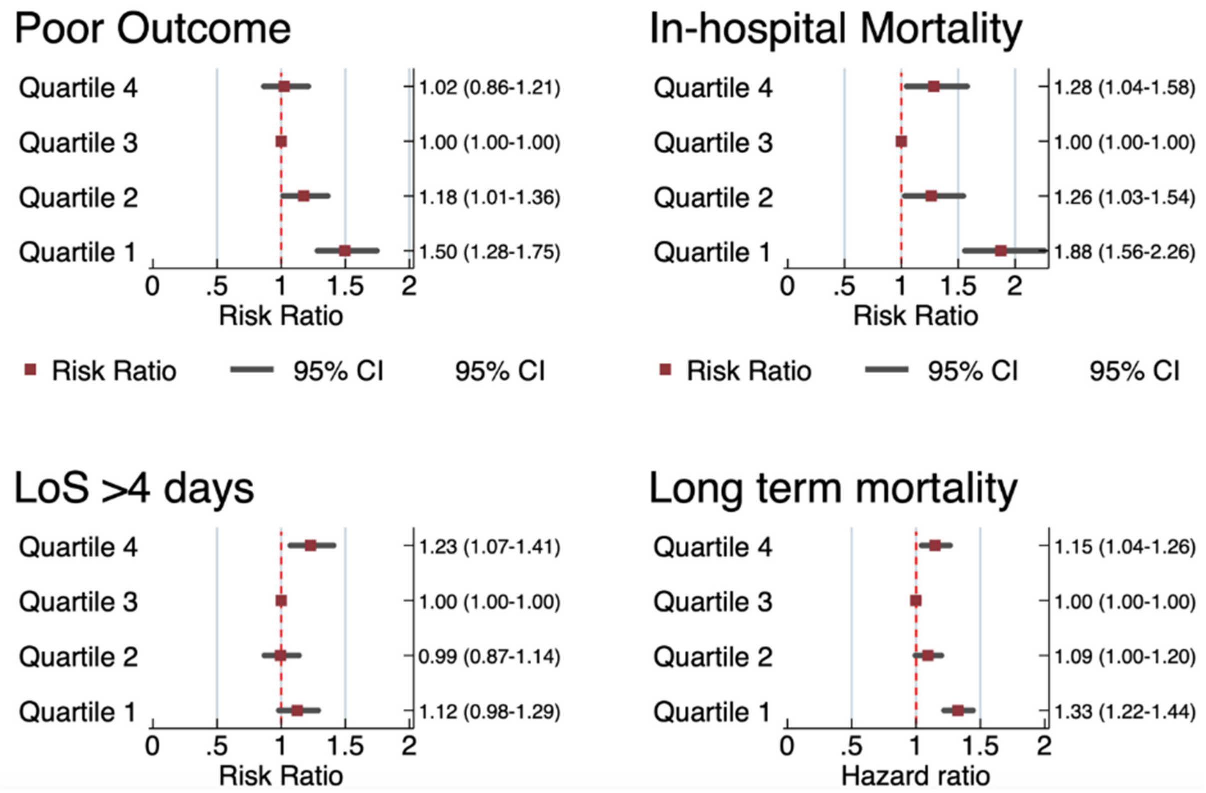

Figure S4. Forest plot assessing the association between serum albumin and clinical outcomes without National Institute of Health Stroke Scale adjustment. The association with serum albumin level and in-hospital mortality, poor functional outcomes (modified Rankin Scale (mRS) score of 3-6) and increased length of stay (>4days). The association of serum albumin and long-term mortality at 5.5 year follow up. The blue line indicates the RR/HR, and the grey area indicates the confidence interval. The red line demonstrates the null effect line. The long-term mortality models were fitted by a Cox regression with adjustments made for age, sex comorbidities (chronic pulmonary disease, atrial fibrillation, cerebrovascular disease, congestive heart disease, heart failure, renal disease, COPD, dementia, diabetes, hypertension, hyperlipidaemia, liver disease, cancer, peptic ulcer disease, peripheral disease and connective tissue disease, pneumonia (aspiration and non-aspiration), anti-platelet and anti-coagulation medication on discharge/admission, OSCP scale, pre-stroke mRS score, serum white blood count, C-reactive protein.

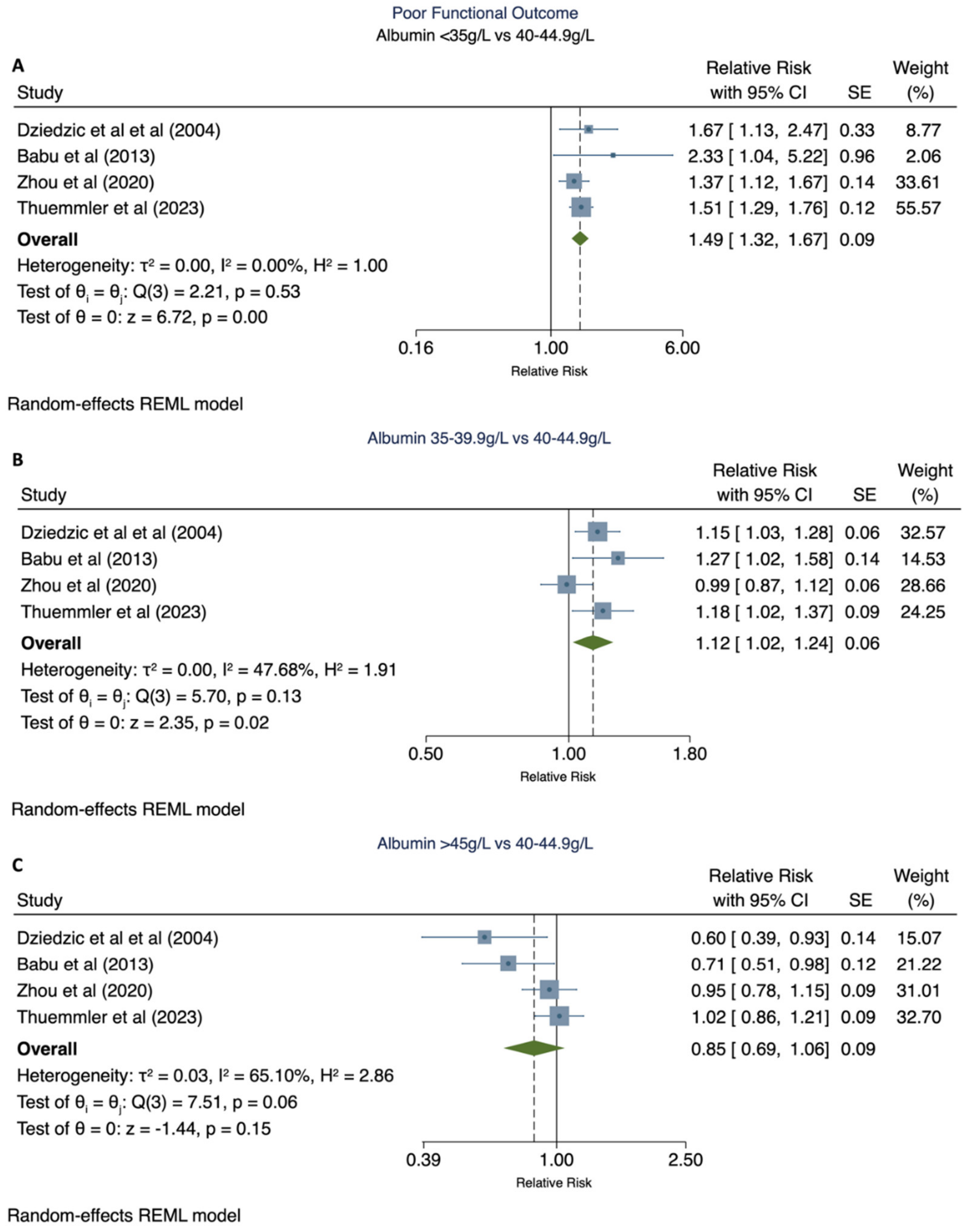

Figure S5. Meta-analysis of the association of categorical serum albumin levels with poor functional outcomes. (A) demonstrates the pooled relative risk of poor functional outcomes for serum albumin levels <35 g/L vs. 40-44 g/L. (B) demonstrates the pooled relative risk of serum albumin levels 35-39.9 g/L vs. 40-44 g/L. (C) demonstrates the relative risk of serum albumin levels >45 g/L vs. 40-44 g/L. CI, confidence interval; SE, standard error; P, *p*-value.

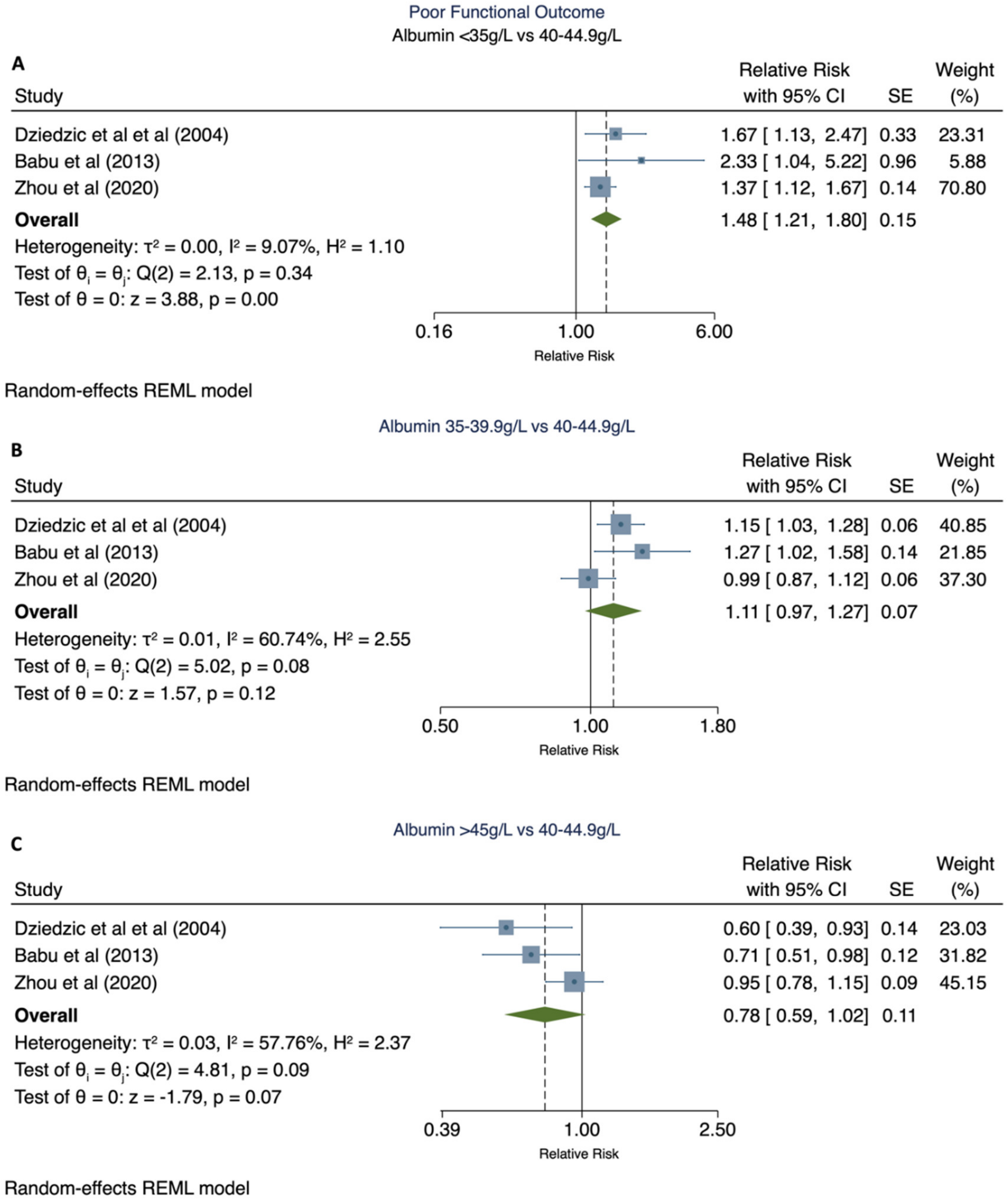

Figure S6. Sensitivity analyses of poor functional outcomes by omitting Thuemmler *et al*., due variation in follow up. (A) demonstrates the pooled relative risk of poor functional outcomes for serum albumin levels <35 g/L vs. 40-44 g/L. (B) demonstrates the pooled relative risk of serum albumin levels 35–39.9 g/L vs. 40-44 g/L. (C) demonstrates serum albumin levels >45 g/L vs. 40-44 g/L. CI, confidence interval; SE, standard error; P, *p*-value.

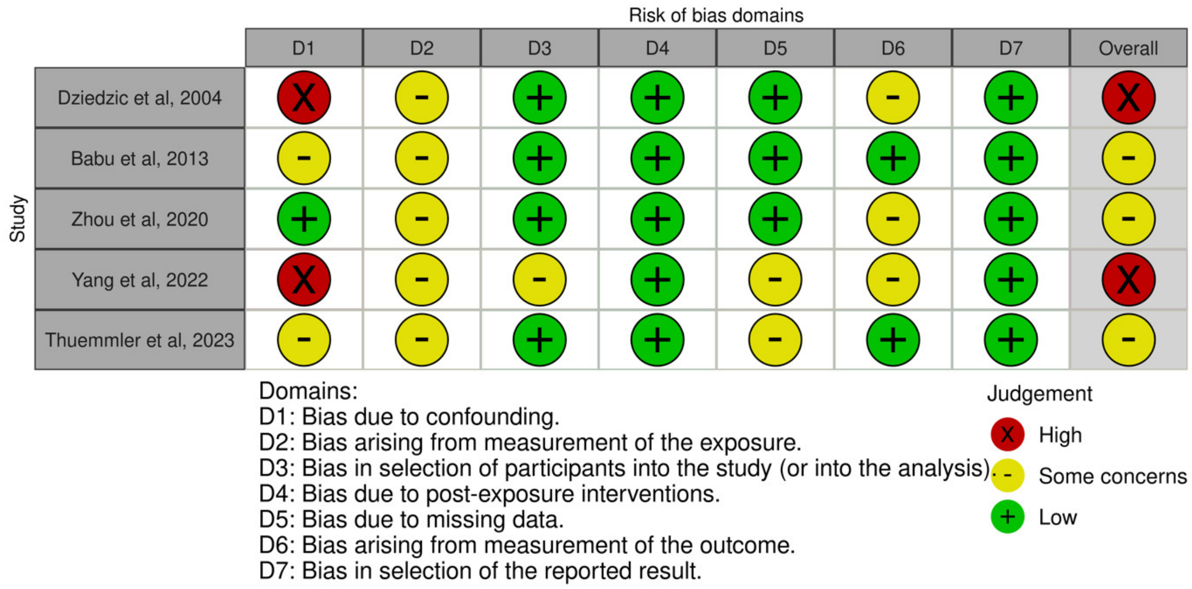

Figure S7. Risk of bias assessment of papers assessing albumin levels and poor functional outcome

Supplemental References

1. Zhou H, Wang A, Meng X, et al. Low serum albumin levels predict poor outcome in patients with acute ischaemic stroke or transient ischaemic attack. *Stroke Vasc Neurol* 2021; 6: 458–466.
2. Gariballa SE, Parker SG, Taub N, et al. Influence of nutritional status on clinical outcome after acute stroke. *Am J Clin Nutr* 1998; 68: 275–281.
3. Carter AM, Catto AJ, Mansfield MW, et al. Predictive Variables for Mortality After Acute Ischemic Stroke. Epub ahead of print 2007. DOI: 10.1161/STROKEAHA.106.474569.
4. Idicula TT, Waje-Andreassen U, Brogger J, et al. Serum Albumin in Ischemic Stroke Patients: The Higher the BetterThe Bergen Stroke Study. *Cerebrovascular Diseases* 2009; 28: 13–17.
5. Lázaro VA, Del Ser Quijano T, Martín RB. Hypoalbuminemia and other prognostic factors of mortality at different time points after ischemic stroke. *Nutr Hosp* 2013; 28: 456–463.
6. Chapter 10: Analysing data and undertaking meta-analyses | Cochrane Training, https://training.cochrane.org/handbook/current/chapter-10 (accessed 3 September 2023).
7. Yang D, Shen J, Huang H, et al. Elevated Albumin to Globulin Ratio on Day 7 is Associated with Improved Function Outcomes in Acute Ischemic Stroke Patients with Intravenous Thrombolysis. Epub ahead of print 2022. DOI: 10.2147/JIR.S347026.
8. Dziedzic T, Slowik A, Szczudlik A. Serum Albumin Level as a Predictor of Ischemic Stroke Outcome. *Stroke*; 35. Epub ahead of print 1 June 2004. DOI: 10.1161/01.STR.0000126609.18735.BE.
9. Babu MS, Kaul S, Dadheech S, et al. Serum albumin levels in ischemic stroke and its subtypes: correlation with clinical outcome. *Nutrition* 2013; 29: 872–875.
